# The genome sequence of the Lesser Yellow Underwing, *Noctua comes *Hübner, 1813

**DOI:** 10.12688/wellcomeopenres.21223.1

**Published:** 2024-04-17

**Authors:** Douglas Boyes, Liam M. Crowley, Finley Hutchinson, Denise C. Wawman, University of Oxford and Wytham Woods Genome Acquisition Lab, Darwin Tree of Life Barcoding collective, Wellcome Sanger Institute Tree of Life Management, Samples and Laboratory team, Wellcome Sanger Institute Scientific Operations: Sequencing Operations, Wellcome Sanger Institute Tree of Life Core Informatics team, Tree of Life Core Informatics collective, Darwin Tree of Life Consortium

**Affiliations:** 1 UK Centre for Ecology & Hydrology, Wallingford, England, UK; 2 Department of Biology, University of Oxford, Oxford, England, UK; 3 University of Exeter, Penryn, England, UK

**Keywords:** Noctua comes, Lesser Yellow Underwing, genome sequence, chromosomal, Lepidoptera

## Abstract

We present a genome assembly from an individual female *Noctua comes* (the Lesser Yellow Underwing; Arthropoda; Insecta; Lepidoptera; Noctuidae). The genome sequence is 540.7 megabases in span. Most of the assembly is scaffolded into 32 chromosomal pseudomolecules, including the W and Z sex chromosomes. The mitochondrial genome has also been assembled and is 15.37 kilobases in length. Gene annotation of this assembly on Ensembl identified 18,001 protein coding genes.

## Species taxonomy

Eukaryota; Opisthokonta; Metazoa; Eumetazoa; Bilateria; Protostomia; Ecdysozoa; Panarthropoda; Arthropoda; Mandibulata; Pancrustacea; Hexapoda; Insecta; Dicondylia; Pterygota; Neoptera; Endopterygota; Amphiesmenoptera; Lepidoptera; Glossata; Neolepidoptera; Heteroneura; Ditrysia; Obtectomera; Noctuoidea; Noctuidae; Noctuinae; Noctuini; *Noctua*; *Noctua comes* Hübner, 1813 (NCBI:txid987992).

## Background

*Noctua comes*, the Lesser Yellow Underwing, is a moth in the family Noctuidae and has the typical fat body and relatively narrow wing seen in this group of moths. Like other yellow underwings, it has forewings in varying shades of brown, and yellow hind wings with a black band towards their trailing edge, but it can be distinguished from the similar Lunar Yellow Underwing *Noctua orbona* by its less clearly defined triangular black marking at the apex of the forewing, and from the Large Yellow Underwing *Noctua pronuba* which lacks the large discal spot seen in *N. comes* ( Skinner & Wilson, 2009).

*Noctua comes* is a common moth, found in most habitats throughout Britain and Ireland ( Waring *et al.*, 2017) and, unlike many moths, its numbers increased between 1968 and 2002 ( Conrad *et al.*, 2006). It has a single generation on the wing from June to October. The larvae feed nocturnally from August to May ( Waring *et al.*, 2017), and, like those of many noctuid moths, are known as cutworms because of their habit of causing damage to plant stems by feeding close to the ground ( Bourner & Cory, 2004). The larvae feed on a wide range of small trees, shrubs and herbaceous plants, including common nettle *Urtica dioica*, broad-leaved dock *Rumex obtusifolius*, foxglove *Digitalis purpurea*, hawthorn *Crataegus monogyna*, blackthorn *Prunus spinosa*, sallow *Salix* spp., bramble *Rubus fruticosus*, broom *Cytisus scoparius*, and heather *Calluna vulgaris* ( Skinner & Wilson, 2009; Waring *et al.*, 2017). Where it has been introduced to North America it can also be a pest of crop plants such as tobacco *Nicotiana* spp. and grape *Vitis* spp. ( Copley & Cannings, 2005; Crolla, 2008) and there have been attempts to find biological methods of control using various viruses ( Bourner & Cory, 2004).

Like many moths, *Noctua comes* is important in the diet of bats ( Mitschunas & Wagner, 2015; Norman *et al.*, 1999). Moths can detect ultrasonic waves and are better at evading Greater Mouse-eared Bat *Myotis myotis* and the Lesser Mouse-eared Bat *Myotis blythii* which have been ringed with a metal and plastic ring positioned on the same limb so that they can rub together, because the frequency of the sound emitted by the rings is closer to the best auditory frequency of the moth than that of the calls of the bats ( Norman *et al.*, 1999).

We present a chromosomally complete genome sequence for *Noctua comes*, based on a female specimen from Wytham Woods, Oxfordshire, UK.

## Genome sequence report

**Figure 1.**
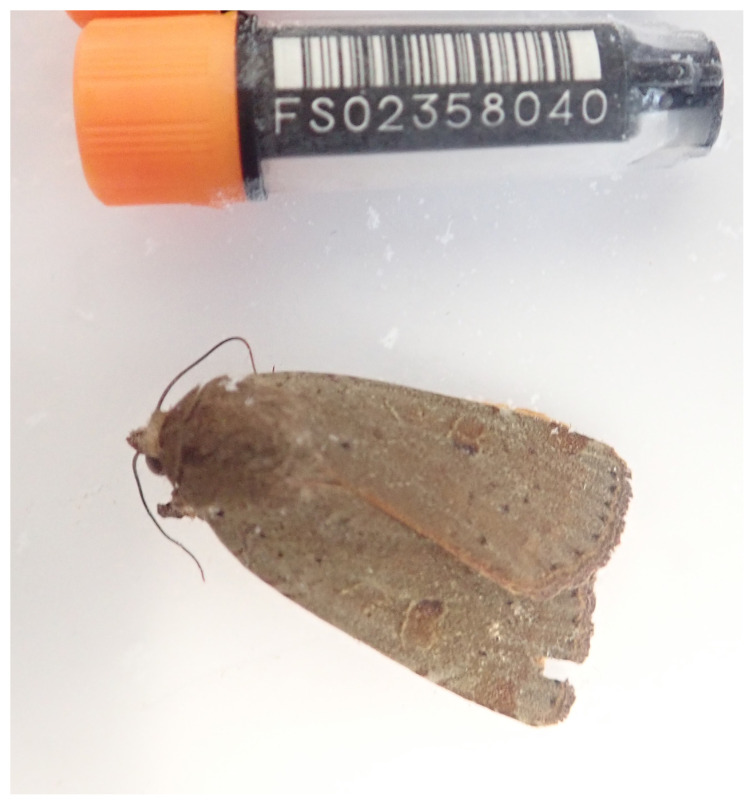
Photograph of the *Noctua comes* (ilNocCome1) specimen used for genome sequencing.

The genome was sequenced from a female *Noctua comes* ( Figure 1) collected from Wytham Woods, Oxfordshire, UK (51.77, –1.34). A total of 43-fold coverage in Pacific Biosciences single-molecule HiFi long reads was generated. Primary assembly contigs were scaffolded with chromosome conformation Hi-C data.

**Figure 2.**
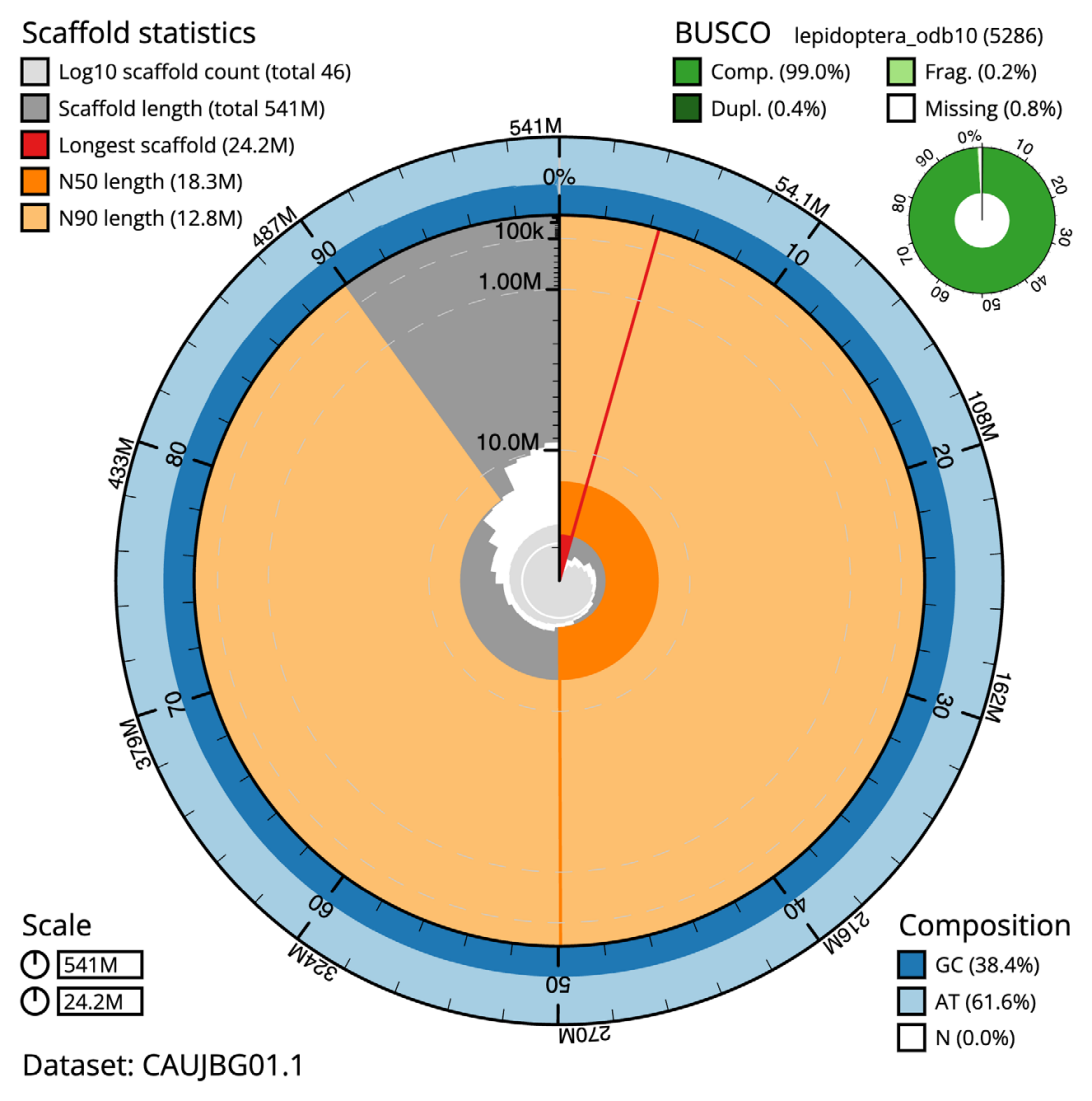
Genome assembly of *Noctua comes*, ilNocCome1.1: metrics. The BlobToolKit snail plot shows N50 metrics and BUSCO gene completeness. The main plot is divided into 1,000 size-ordered bins around the circumference with each bin representing 0.1% of the 540,735,880 bp assembly. The distribution of scaffold lengths is shown in dark grey with the plot radius scaled to the longest scaffold present in the assembly (24,202,804 bp, shown in red). Orange and pale-orange arcs show the N50 and N90 scaffold lengths (18,283,226 and 12,831,000 bp), respectively. The pale grey spiral shows the cumulative scaffold count on a log scale with white scale lines showing successive orders of magnitude. The blue and pale-blue area around the outside of the plot shows the distribution of GC, AT and N percentages in the same bins as the inner plot. A summary of complete, fragmented, duplicated and missing BUSCO genes in the lepidoptera_odb10 set is shown in the top right. An interactive version of this figure is available at https://blobtoolkit.genomehubs.org/view/CAUJBG01/dataset/CAUJBG01/snail.

**Figure 3.**
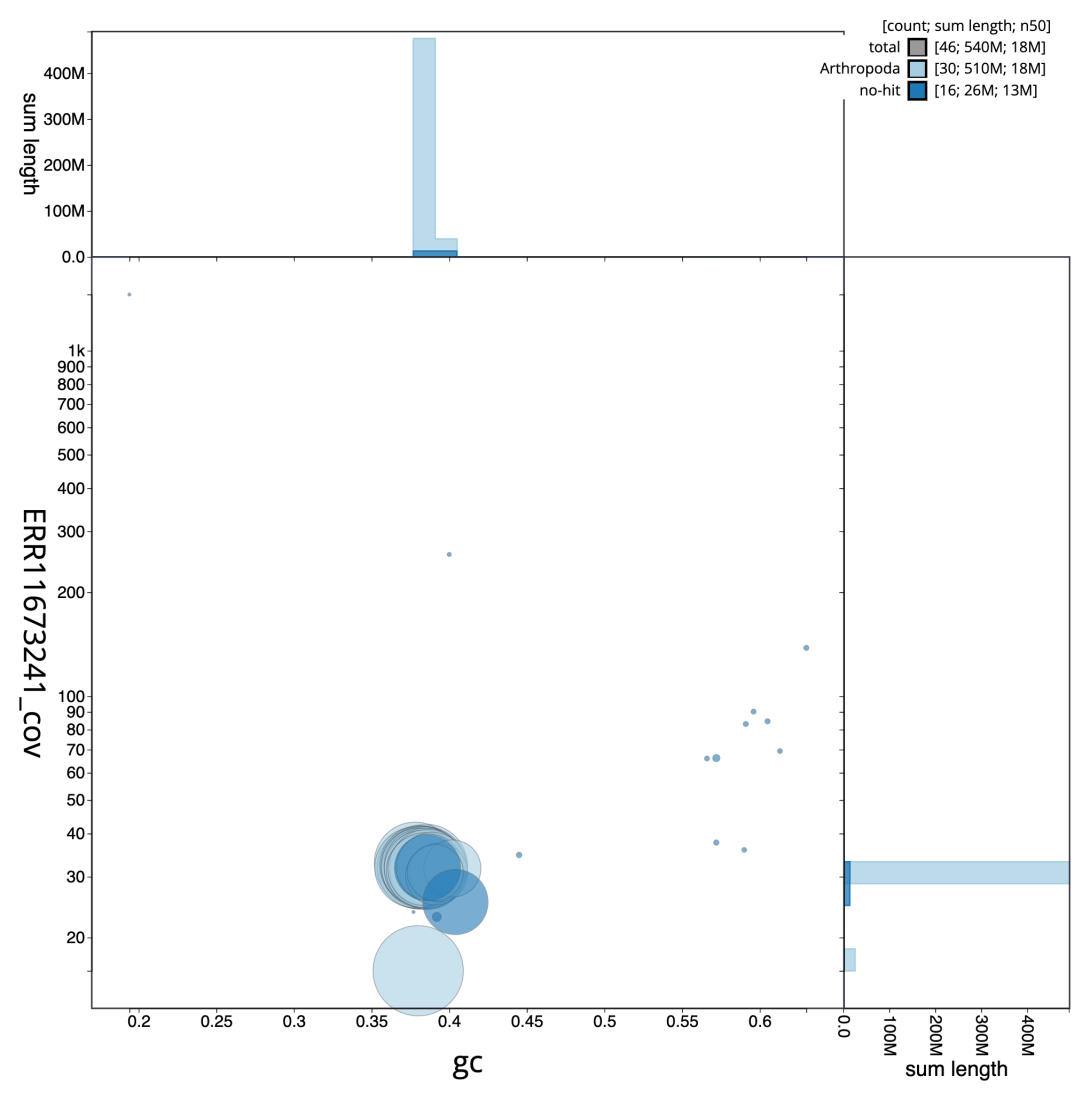
Genome assembly of *Noctua comes*, ilNocCome1.1: BlobToolKit GC-coverage plot. Sequences are coloured by phylum. Circles are sized in proportion to sequence length. Histograms show the distribution of sequence length sum along each axis. An interactive version of this figure is available at https://blobtoolkit.genomehubs.org/view/CAUJBG01/dataset/CAUJBG01.1/blob.

**Figure 4.**
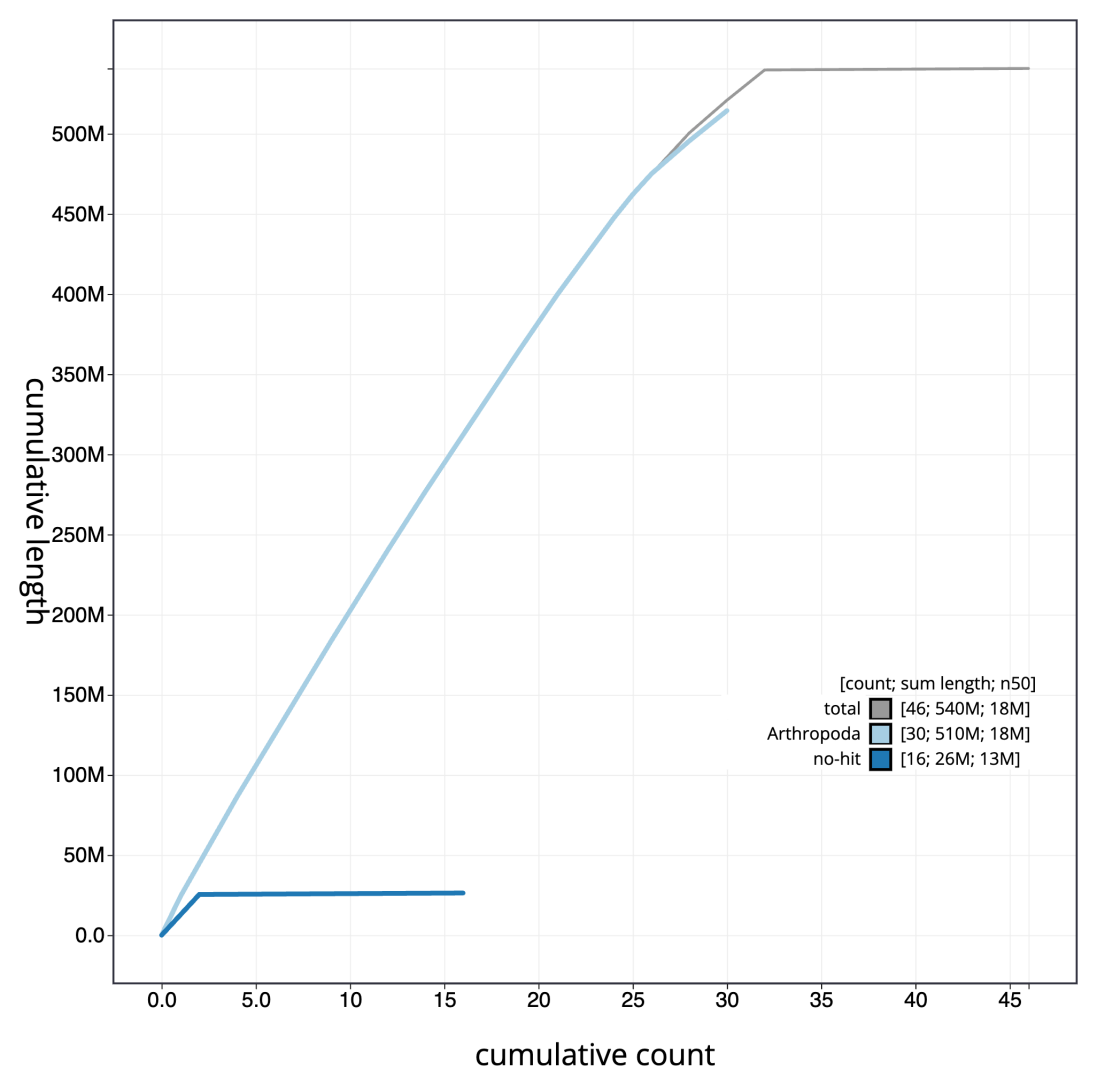
Genome assembly of *Noctua comes*, ilNocCome1.1: BlobToolKit cumulative sequence plot. The grey line shows cumulative length for all sequences. Coloured lines show cumulative lengths of sequences assigned to each phylum using the buscogenes taxrule. An interactive version of this figure is available at https://blobtoolkit.genomehubs.org/view/CAUJBG01/dataset/CAUJBG01/cumulative.

**Figure 5.**
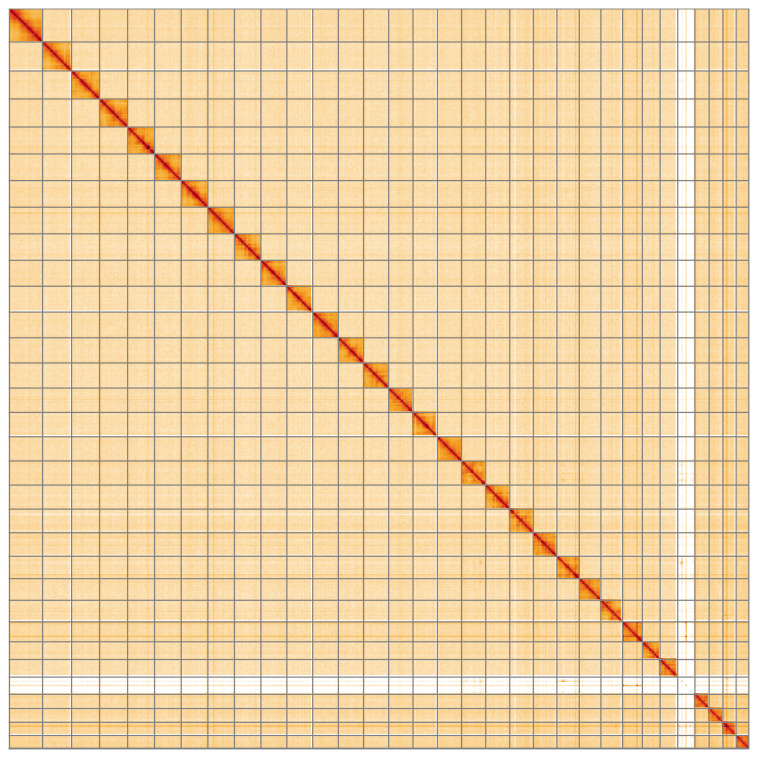
Genome assembly of *Noctua comes*, ilNocCome1.1: Hi-C contact map of the ilNocCome1.1 assembly, visualised using HiGlass. Chromosomes are shown in order of size from left to right and top to bottom. An interactive version of this figure may be viewed at https://genome-note-higlass.tol.sanger.ac.uk/l/?d=JlNOMLnKTpGGbP0ewYF8cA.

The final assembly has a total length of 540.7 Mb in 45 sequence scaffolds with a scaffold N50 of 18.3 Mb ( Table 1). The snail plot in Figure 2 provides a summary of the assembly statistics, while the distribution of assembly scaffolds on GC proportion and coverage is shown in Figure 3. The cumulative assembly plot in Figure 4 shows curves for subsets of scaffolds assigned to different phyla. Most (99.83%) of the assembly sequence was assigned to 32 chromosomal-level scaffolds, representing 30 autosomes and the W and Z sex chromosomes. Chromosome-scale scaffolds confirmed by the Hi-C data are named in order of size ( Figure 5; Table 2). Chromosome Z and W were identified by read coverage statistics. While not fully phased, the assembly deposited is of one haplotype. Contigs corresponding to the second haplotype have also been deposited. The mitochondrial genome was also assembled and can be found as a contig within the multifasta file of the genome submission.

**Table 1.** Genome data for *Noctua comes*, ilNocCome1.1.

Project accession data		
Assembly identifier	ilNocCome1.1	
Species	*Noctua comes*	
Specimen	ilNocCome1	
NCBI taxonomy ID	987992	
BioProject	PRJEB64088	
BioSample ID	SAMEA7701458	
Isolate information	ilNocCome1, female: abdomen (DNA sequencing) ilNocCome2: head (Hi-C sequencing), abdomen (RNA sequencing)	
Assembly metrics *		*Benchmark*
Consensus quality (QV)	71.2	*≥ 50*
*k*-mer completeness	100.0%	*≥ 95%*
BUSCO **	C:99.0%[S:98.6%,D:0.4%], F:0.2%,M:0.8%,n:5,286	*C ≥ 95%*
Percentage of assembly mapped to chromosomes	99.83%	*≥ 95%*
Sex chromosomes	WZ	*localised homologous pairs*
Organelles	Mitochondrial genome: 15.37 kb	*complete single alleles*
Raw data accessions		
PacificBiosciences SEQUEL II	ERR11673241	
Hi-C Illumina	ERR11679406	
PolyA RNA-Seq Illumina	ERR12245580	
Genome assembly		
Assembly accession	GCA_963082995.1	
*Accession of alternate haplotype*	GCA_963082645.1	
Span (Mb)	540.7	
Number of contigs	50	
Contig N50 length (Mb)	18.3	
Number of scaffolds	45	
Scaffold N50 length (Mb)	18.3	
Longest scaffold (Mb)	24.2	
Genome annotation		
Number of protein-coding genes	18,001	
Number of gene transcripts	18,210	

* Assembly metric benchmarks are adapted from column VGP-2020 of “Table 1: Proposed standards and metrics for defining genome assembly quality” from Rhie *et al.* (2021). ** BUSCO scores based on the lepidoptera_odb10 BUSCO set using version 5.3.2. C = complete [S = single copy, D = duplicated], F = fragmented, M = missing, n = number of orthologues in comparison. A full set of BUSCO scores is available at https://blobtoolkit.genomehubs.org/view/CAUJBG01/dataset/CAUJBG01/busco.

**Table 2.** Chromosomal pseudomolecules in the genome assembly of *Noctua comes*, ilNocCome1.

INSDC accession	Chromosome	Length (Mb)	GC%
OY720267.1	1	21.14	38.0
OY720268.1	2	20.47	38.5
OY720269.1	3	20.46	38.0
OY720270.1	4	19.67	38.0
OY720271.1	5	19.47	38.0
OY720272.1	6	19.45	38.5
OY720273.1	7	19.42	38.0
OY720274.1	8	19.3	38.0
OY720275.1	9	18.98	38.0
OY720276.1	10	18.84	38.0
OY720277.1	11	18.78	38.0
OY720278.1	12	18.4	38.5
OY720279.1	13	18.28	38.0
OY720280.1	14	17.81	38.0
OY720281.1	15	17.74	38.5
OY720282.1	16	17.68	38.5
OY720283.1	17	17.63	38.5
OY720284.1	18	17.56	38.5
OY720285.1	19	17.21	38.5
OY720286.1	20	17.2	38.5
OY720287.1	21	16.37	38.5
OY720288.1	22	15.77	38.5
OY720289.1	23	15.72	38.5
OY720290.1	24	14.58	39.0
OY720291.1	25	12.96	38.5
OY720292.1	26	12.83	38.5
OY720294.1	27	10.36	39.0
OY720295.1	28	10.07	39.5
OY720296.1	29	9.62	40.0
OY720297.1	30	9.26	39.0
OY720293.1	W	12.58	40.5
OY720266.1	Z	24.2	38.0
OY720298.1	MT	0.02	19.5

The estimated Quality Value (QV) of the final assembly is 71.2 with *k*-mer completeness of 100.0%, and the assembly has a BUSCO v5.3.2 completeness of 99.0% (single = 98.6%, duplicated = 0.4%), using the lepidoptera_odb10 reference set ( *n* = 5,286).

Metadata for specimens, barcode results, spectra estimates, sequencing runs, contaminants and pre-curation assembly statistics are given at https://links.tol.sanger.ac.uk/species/987992.

## Genome annotation report

The *Noctua comes* genome assembly (GCA_963082995.1) was annotated at the European Bioinformatics Institute (EBI) on Ensembl Rapid Release. The resulting annotation includes 18,210 transcribed mRNAs from 18,001 protein-coding genes ( Table 1; https://rapid.ensembl.org/Noctua_comes_GCA_963082995.1/Info/Index).

## Methods

### Sample acquisition and nucleic acid extraction

A specimen of *Noctua comes* (specimen ID Ox000594, ToLID ilNocCome1) was collected from Wytham Woods, Oxfordshire (biological vice-county Berkshire), UK (latitude 51.77, longitude –1.34) on 2020-07-05 using a light trap. The specimen was collected and identified by Douglas Boyes (University of Oxford). The specimen used for Hi-C and RNA sequencing (specimen ID Ox003031, ToLID ilNocCome2) was collected from the same location on 2022-07-22, also using a light trap. The specimen was collected by Liam Crowley (University of Oxford) and Finley Hutchinson (University of Exeter), and identified by Finley Hutchinson. The specimens were stored, handled and delivered on dry ice.

The workflow for high molecular weight (HMW) DNA extraction at the Wellcome Sanger Institute (WSI) includes a sequence of core procedures: sample preparation; sample homogenisation, DNA extraction, fragmentation, and clean-up. In sample preparation, the ilNocCome1 sample was weighed and dissected on dry ice ( Jay *et al.*, 2023). Tissue from the abdomen was homogenised using a PowerMasher II tissue disruptor ( Denton *et al.*, 2023a). HMW DNA was extracted using the Automated MagAttract v1 protocol ( Sheerin *et al.*, 2023). DNA was sheared into an average fragment size of 12–20 kb in a Megaruptor 3 system with speed setting 30 ( Todorovic *et al.*, 2023). Sheared DNA was purified by solid-phase reversible immobilisation ( Strickland *et al.*, 2023): in brief, the method employs a 1.8X ratio of AMPure PB beads to sample to eliminate shorter fragments and concentrate the DNA. The concentration of the sheared and purified DNA was assessed using a Nanodrop spectrophotometer and Qubit Fluorometer and Qubit dsDNA High Sensitivity Assay kit. Fragment size distribution was evaluated by running the sample on the FemtoPulse system.

RNA was extracted from abdomen tissue of ilNocCome1 in the Tree of Life Laboratory at the WSI using the RNA Extraction: Automated MagMax™ *mir*Vana protocol ( do Amaral *et al.*, 2023). The RNA concentration was assessed using a Nanodrop spectrophotometer and a Qubit Fluorometer using the Qubit RNA Broad-Range Assay kit. Analysis of the integrity of the RNA was done using the Agilent RNA 6000 Pico Kit and Eukaryotic Total RNA assay.

Protocols developed by the WSI Tree of Life laboratory are publicly available on protocols.io ( Denton *et al.*, 2023b).

### Sequencing

Pacific Biosciences HiFi circular consensus DNA sequencing libraries were constructed according to the manufacturers’ instructions. Poly(A) RNA-Seq libraries were constructed using the NEB Ultra II RNA Library Prep kit. DNA and RNA sequencing was performed by the Scientific Operations core at the WSI on Pacific Biosciences SEQUEL II (HiFi) and Illumina NovaSeq 6000 (RNA-Seq) instruments. Hi-C data were also generated from head tissue of ilNocCome2 using the Arima2 kit and sequenced on the Illumina NovaSeq 6000 instrument.

### Genome assembly, curation and evaluation

Assembly was carried out with Hifiasm ( Cheng *et al.*, 2021) and haplotypic duplication was identified and removed with purge_dups ( Guan *et al.*, 2020). The assembly was then scaffolded with Hi-C data ( Rao *et al.*, 2014) using YaHS ( Zhou *et al.*, 2023). The assembly was checked for contamination and corrected as described previously ( Howe *et al.*, 2021). Manual curation was performed using HiGlass ( Kerpedjiev *et al.*, 2018) and PretextView ( Harry, 2022). The mitochondrial genome was assembled using MitoHiFi ( Uliano-Silva *et al.*, 2023), which runs MitoFinder ( Allio *et al.*, 2020) or MITOS ( Bernt *et al.*, 2013) and uses these annotations to select the final mitochondrial contig and to ensure the general quality of the sequence.

A Hi-C map for the final assembly was produced using bwa-mem2 ( Vasimuddin *et al.*, 2019) in the Cooler file format ( Abdennur & Mirny, 2020). To assess the assembly metrics, the *k*-mer completeness and QV consensus quality values were calculated in Merqury ( Rhie *et al.*, 2020). This work was done using Nextflow ( Di Tommaso *et al.*, 2017) DSL2 pipelines “sanger-tol/readmapping” ( Surana *et al.*, 2023a) and “sanger-tol/genomenote” ( Surana *et al.*, 2023b). The genome was analysed within the BlobToolKit environment ( Challis *et al.*, 2020) and BUSCO scores ( Manni *et al.*, 2021; Simão *et al.*, 2015) were calculated.

Table 3 contains a list of relevant software tool versions and sources.

**Table 3.** Software tools: versions and sources.

Software tool	Version	Source
BlobToolKit	4.2.1	https://github.com/blobtoolkit/blobtoolkit
BUSCO	5.3.2	https://gitlab.com/ezlab/busco
Hifiasm	0.19.5-r587	https://github.com/chhylp123/hifiasm
HiGlass	1.11.6	https://github.com/higlass/higlass
Merqury	MerquryFK	https://github.com/thegenemyers/MERQURY.FK
MitoHiFi	3	https://github.com/marcelauliano/MitoHiFi
PretextView	0.2	https://github.com/wtsi-hpag/PretextView
purge_dups	1.2.5	https://github.com/dfguan/purge_dups
sanger-tol/genomenote	v1.0	https://github.com/sanger-tol/genomenote
sanger-tol/readmapping	1.1.0	https://github.com/sanger-tol/readmapping/tree/1.1.0
YaHS	1.2a.2	https://github.com/c-zhou/yahs

### Genome annotation

The BRAKER2 pipeline ( Brůna *et al.*, 2021) was used in the default protein mode to generate annotation for the *Noctua comes* assembly (GCA_963082995.1) in Ensembl Rapid Release at the EBI.

### Wellcome Sanger Institute – Legal and Governance

The materials that have contributed to this genome note have been supplied by a Darwin Tree of Life Partner. The submission of materials by a Darwin Tree of Life Partner is subject to the **‘Darwin Tree of Life Project Sampling Code of Practice’**, which can be found in full on the Darwin Tree of Life website here. By agreeing with and signing up to the Sampling Code of Practice, the Darwin Tree of Life Partner agrees they will meet the legal and ethical requirements and standards set out within this document in respect of all samples acquired for, and supplied to, the Darwin Tree of Life Project.

Further, the Wellcome Sanger Institute employs a process whereby due diligence is carried out proportionate to the nature of the materials themselves, and the circumstances under which they have been/are to be collected and provided for use. The purpose of this is to address and mitigate any potential legal and/or ethical implications of receipt and use of the materials as part of the research project, and to ensure that in doing so we align with best practice wherever possible. The overarching areas of consideration are:

• Ethical review of provenance and sourcing of the material

• Legality of collection, transfer and use (national and international)

Each transfer of samples is further undertaken according to a Research Collaboration Agreement or Material Transfer Agreement entered into by the Darwin Tree of Life Partner, Genome Research Limited (operating as the Wellcome Sanger Institute), and in some circumstances other Darwin Tree of Life collaborators.

## Data Availability

European Nucleotide Archive: *Noctua comes* (lesser yellow underwing). Accession number PRJEB64088; https://identifiers.org/ena.embl/PRJEB64088 ( Wellcome Sanger Institute, 2023). The genome sequence is released openly for reuse. The *Noctua comes* genome sequencing initiative is part of the Darwin Tree of Life (DToL) project. All raw sequence data and the assembly have been deposited in INSDC databases. Raw data and assembly accession identifiers are reported in Table 1.

## Author information

Members of the University of Oxford and Wytham Woods Genome Acquisition Lab are listed here: https://doi.org/10.5281/zenodo.7125292.

Members of the Darwin Tree of Life Barcoding collective are listed here: https://doi.org/10.5281/zenodo.4893703.

Members of the Wellcome Sanger Institute Tree of Life Management, Samples and Laboratory team are listed here: https://doi.org/10.5281/zenodo.10066175.

Members of Wellcome Sanger Institute Scientific Operations: Sequencing Operations are listed here: https://doi.org/10.5281/zenodo.10043364.

Members of the Wellcome Sanger Institute Tree of Life Core Informatics team are listed here: https://doi.org/10.5281/zenodo.10066637.

Members of the Tree of Life Core Informatics collective are listed here: https://doi.org/10.5281/zenodo.5013541.

Members of the Darwin Tree of Life Consortium are listed here: https://doi.org/10.5281/zenodo.4783558.
