# Supplementary material for: The genome sequence of the Lesser Yellow Underwing, Noctua comes Hübner, 1813
Source: Wellcome Open Res. 2024 Apr 17;9:200. [Version 1] doi: 10.12688/wellcomeopenres.21223.1 (PMC11347922; doi:10.12688/wellcomeopenres.21223.1)
Supplement: Peer review history

# Reviewer report: Reviewer response for version 1

**DOI:** 10.21956/wellcomeopenres.23470.r92023

**Published:** 2024-08-26

**Authors:** Yuttapong Thawornwattana

**Affiliations:** 1 Department of Organismic and Evolutionary Biology, Harvard University, Cambridge, Massachusetts, USA

Boyes *et al* present a 540.7-Mb chromosomally complete assembly of *Noctua comes* Hübner, 1813 (Lepidoptera: Noctuidae) based on a female specimen collected from Wytham Woods in Oxfordshire, England. 

 The assembly is of high quality and will be useful to the research community.  It contains 30 autosomes, the Z and W chromosomes and the mitochondrial genome.  It would be helpful to label chromosomes in the Hi-C contact map (fig 5).  Some chromosomes seem to have a strange pattern of genome-wide contact, e.g. second, fifth and eights from last.  Could this be an artefact of the assembly?  Adding information about the expected chromosome number, e.g. from cytology studies, would be useful.

Are sufficient details of methods and materials provided to allow replication by others?

Yes

Is the rationale for creating the dataset(s) clearly described?

Yes

Are the datasets clearly presented in a useable and accessible format?

Yes

Are the protocols appropriate and is the work technically sound?

Yes

Reviewer Expertise:

evolutionary genomics, phylogenetics, insect evolution

I confirm that I have read this submission and believe that I have an appropriate level of expertise to confirm that it is of an acceptable scientific standard.

# Reviewer report: Reviewer response for version 1

**DOI:** 10.21956/wellcomeopenres.23470.r90402

**Published:** 2024-08-07

**Authors:** Maria Nilsson

**Affiliations:** 1 Senckenberg Biodiversity and Climate Research Centre, Frankfurt am Main, Germany

The genome of *Noctua comes,* the lesser yellow underwing moth, as been assembled into a highly contiguous assembly.

 Two individuals of *Noctua* comes was used to generate the assembly, both captured using a light trap at the same location. One individual was used for long read sequencing and a second one for RNA-sequencing and HiC.

 The QV is 71.2 and BUSCO score of 99.0% which indicate a very complete assembly. The contig N50 is 18.3Mb and it is assembled into 45 scaffolds. The genome assembly is 540Mb large and the transcriptome analysis identified 18,001 genes.

 I have three minor complaints about the readability in the introduction/Background:

 1) the sentence "It has a single generation on the wing from June to October." does not make sense to me. Please improve if necessary.

 2) The sentence where the three species of moths are described. This is the second sentence in the Background starting with "Like other yellow underwings....". This is a very long and complex sentence and can likely be shortened.

 3) the sentence in the third paragraph starting with "Moths can detect ultrasonic waves and are... ", seems irrelevant to the species *Noctua comes*.

Are sufficient details of methods and materials provided to allow replication by others?

Yes

Is the rationale for creating the dataset(s) clearly described?

Yes

Are the datasets clearly presented in a useable and accessible format?

Yes

Are the protocols appropriate and is the work technically sound?

Yes

Reviewer Expertise:

phylogenomics, transposable elements, mitogenomics

I confirm that I have read this submission and believe that I have an appropriate level of expertise to confirm that it is of an acceptable scientific standard.
